# Validity and Reliability of a Photoelectric Cells System for the Evaluation of Change of Direction and Lateral Jumping Abilities in Collegiate Basketball Athletes

**DOI:** 10.3390/jfmk5030055

**Published:** 2020-07-22

**Authors:** Giancarlo Condello, Chutimon Khemtong, Yi-Hua Lee, Chi-Hsien Chen, Mauro Mandorino, Enrico Santoro, Chiang Liu, Antonio Tessitore

**Affiliations:** 1 Graduate Institute of Sports Training, Institute of Sports Sciences, University of Taipei, 101 Zhongcheng Rd. Section 2, Shilin District, Taipei 111, Taiwan; tmedmet.md_sci3@hotmail.com; 2 Department of Ball Sports, University of Taipei, 101 Zhongcheng Rd. Section 2, Shilin District, Taipei 111, Taiwan; lykllyy@utaipei.edu.tw; 3 Institute of Sports Equipment Technology, University of Taipei, 101 Zhongcheng Rd. Section 2, Shilin District, Taipei 111, Taiwan; s840410s@gmail.com (C.-H.C.); chiangliu1974@yahoo.com.tw (C.L.); 4 Department of Movement, Human and Health Sciences, University of Rome Foro Italico, Piazza Lauro De Bosis 6, 00135 Roma, Italy; mauromandorino@gmail.com (M.M.); enry9521@gmail.com (E.S.); antonio.tessitore@uniroma4.it (A.T.)

**Keywords:** optojump system, concurrent validity, internal consistency reliability, sport performance, field-based approach

## Abstract

The validity and reliability of the Optojump system were investigated for jumping height and flight time in vertical jump tests. Conversely, the purpose of the present study was to investigate the validity and reliability of the Optojump system for measuring contact time and lateral displacement in change of direction and lateral jump tests. Thirty basketball collegiate athletes were tested on two 10 m sprints with a 60° (COD60) or 180° (COD180) change of direction, lateral controlled (CLRJ) and maximal (MLRJ) rebound jump, and lateral countermovement (LCMJ) and squat (LSJ) jump with the concomitant use of two force plates and the Optojump system for the measurement of contact time in COD60, COD180, CLRJ, MLRJ, and lateral jumping distance in all the lateral jump tests. Almost perfect coefficients (r ≥ 0.95) emerged for contact time in COD60, COD180, CLRJ, MLRJ, although a systematic bias was found for COD60 (−0.01 s). Good-to-excellent reliability was found for almost all the measurements of contact time and lateral jumping distance for change of direction and lateral jump tests. Therefore, the use of Optojump system for testing change of direction and lateral jumping abilities should be executed with caution, avoiding misinterpretation of data.

## 1. Introduction

Change of direction and jumping abilities represent an expression of the reactive strength, which has been defined as “the ability to quickly switch from an eccentric contraction to a concentric contraction” [1]. The coupling of an eccentric and concentric contraction refers to the stretch-shortening cycle [2]. Both change of direction and jumping abilities rely on the stretch-shortening cycle. Change of direction is a preplanned action and a critical performance determinant in many team sports. It refers to the ability to decelerate (i.e., eccentric component) in the shortest time and quickly re-accelerate (i.e., concentric component) in a new direction while running or sprinting [3,4,5]. The jumping ability can be exerted in a vertical, horizontal, or lateral direction or their combination. Since the nature of the team sports performance is characterized by multidirectional movements, testing athletes under different sport-related performances could be used to ascertain training responses and adaptations, to monitor fatigue and recovery status [6,7,8], and to recognize weaknesses in young athletes and better address talent development [9,10]. Considering that athletes could be tested using a laboratory- and/or a field-based approach, the most suitable and practical approach should be decided based on availability of instruments, time, space, and money.

The use of the force plate in a laboratory setting is considered the “gold standard” [11,12,13] for the measurements of kinetic variables, such as ground reaction force and contact time, hence it is widely used for the evaluation of the jumping ability. However, the force plate still maintains some limitations due to the high cost, the heavy weight and voluminous shape, the installation procedures, the limited portable use, and the experimental procedures [13]. In fact, whilst the force plate installation is permanent or semi-permanent in a laboratory setting, the difficulty to embed it into the floor and the high cost of this operation strongly limit its use on the field in a setting reproducing real movements [13]. Therefore, alternative solutions have been proposed for field-based assessments, such as yardsticks [14], linear position transducers, contact mats [15], and photoelectric cells. The latter (i.e., Optojump photoelectric system) became predominant due to several advantages, such as easy to transport and handle, more suitable for portable applications, and relatively cost effective. In particular, the great advantage of this system is that the two parallel bars (receiver and transmitter units) can be positioned at the floor level of several sport surfaces (except sand), hence maintaining the regular athlete–surface interaction and the content validity [13]. Being commonly used by researchers and practitioners for sport performance investigation, the validity and reliability of the Optojump system has been previously investigated for the evaluation of the vertical jumping ability (i.e., squat and countermovement jump) using the parameters flight time and jump height [12,13,16,17]. However, the Optojump system could also be set up for testing athletes under different movement patterns, particularly if a modular configuration with a longer pathway (i.e., >1 m) is applied. Together with the flight time, the Optojump system can provide other functional parameters of the gait analysis, such as the contact time and displacement. In this regard, these parameters could be used to investigate the change of direction and lateral jumping abilities in team sports athletes. However, to the authors’ knowledge, no studies have attempted to evaluate the validity and reliability of the Optojump system for the measurement of contact time and displacement using tests exerted in directions different from the vertical one. Therefore, the first purpose of this study was to investigate the concurrent validity and internal consistency reliability of the Optojump system for the measurement of the contact time during change of direction and lateral jumping abilities. The second purpose of this study was to investigate the internal consistency reliability of the Optojump system for the evaluation of lateral jumping distance. In light of previous literature demonstrating the validity and reliability of the Optojump system in estimating jumping height during vertical jump modalities [12,13,16,17], it was hypothesized that the Optojump system could be also a valid and reliable instrument for measuring contact time in change of direction and lateral jumping abilities and for measuring lateral displacement.

## 2. Materials and Methods

This study was approved by the University of Taipei Institutional Review Board (Taipei, Taiwan, reference number: IRB-2018-093) and was conducted in accordance with the Declaration of Helsinki. All participants were recruited through flyers and presentation of the study inside the university and gave their informed written consent.

### 2.1. Participants

Thirty male and female collegiate athletes (25 males: height = 179.7 ± 7.4 cm; body mass = 76.3 ± 10.5 kg; 5 females: height = 162.9 ± 7.0 cm; body mass = 58.4 ± 9 kg) were recruited to participate in this study being eligible in accordance to the following inclusion criteria: (a) age 18–25 years; (b) experience of basketball practice for at least five years; (c) absence of known cardiovascular, pulmonary, metabolic, bone, or joint diseases; (d) no smoking; (e) no muscle and joint injuries during the last six months. For each participant, the preferred stance leg (SL) was identified as the stance leg used to jump off when performing a right-handed running basketball lay-up [18], whilst the opposite leg was declared as the leg used to kick a ball (KL). Participants were required to avoid exercising during the 72 h prior to each experimental session and to abstain from alcohol and caffeine consumption during the 12 h prior to each experimental session.

### 2.2. Procedures

Participants reported to the laboratory on three occasions separated by a 72 h resting period. During the first experimental session, participants were familiarized with all instructions to perform the tests. Moreover, height (cm) and body mass (kg) were measured to the nearest decimal using a Jenix DS-102 stadiometer (Dong Sahn Jenix Co., Ltd., Seoul, Korea). Leg length was measured using a retractable tape from the greater trochanter to the lateral malleolus by the same experimenter. During the second and the third experimental session, participants performed the change of direction and the lateral jump tests, in a randomized order. All participants used basketball shoes to reduce the variability given by the use of different type of sport shoes. Before each experimental session, participants completed a standardized warm up involving 3 min of jogging on a treadmill followed by dynamic stretching, squats, frontal and lateral lunges, submaximal jumps (in vertical, frontal, and horizontal directions), short accelerations, directional changes, and submaximal trials of the tests.

The experimental protocol consisted of a series of change of direction and lateral jump tests executed in a laboratory setting with the simultaneous use of two adjacent embedded force plates (60 × 90 cm; BMS 600900 OPTIMA^TM^ Biomechanics Measurement Series, AMTI, Watertown, MA, USA) with a sampling rate of 2400 Hz and an Optojump photoelectric system (OptojumpNext, Microgate, Bolzano, Italy) placed beside the force plates (Figures S1–S3). The change of direction ability was tested under two conditions: (1) a 10 m sprint with a 60° change of direction at 5 m (COD60) in which participants were required to sprint forward for 5 m, make a COD while on the force plate and sprint for another 5 m [19]; (2) a 10 m shuttle sprint with a 180° change of direction at 5 m (COD180) in which participants were required to sprint forward for 5 m, make a COD while on the force plates and sprint back for another 5 m. Participants were instructed to push their foot on an area of the force plate near the middle highlighted by an X. The lateral jumping ability was tested under four conditions: (1) controlled lateral rebound jump (CLRJ); (2) maximal lateral rebound jump (MLRJ); (3) lateral countermovement jump (LCMJ); (4) and lateral squat jump (LSJ). Participants were asked to keep their hands on their hips to eliminate arm swing and to stand on a force plate with a single leg at a distance of 1 m from the middle of the second force plate. For CLRJ, participants were required to jump laterally with one leg (e.g., right leg), rebound (pushing on the second force plate) with the other leg (e.g., left leg) as fast as possible coming back and landing on the starting point with the starting leg. The instantaneous video analysis provided by the Optojump system software was used to ascertain that participants returned to the starting point. For MLRJ, participants were required to jump laterally with one leg (e.g., right leg), rebound (pushing on the second force plate) with the other leg (e.g., left leg) as fast as possible but trying to reach the maximal lateral jumping distance (i.e., as far as possible) landing on the starting leg [20]. They were instructed to push in the middle of the force plate highlighted by an X. Controlled and maximal jumps were chosen in order to investigate the rebound phase when participants had to control the movement or to exert their maximal effort. For LCMJ and LSJ, participants were required to jump laterally with one leg with and without a countermovement, respectively. For the LCMJ, participants were required to bend their knee and self-select the amplitude of the countermovement to avoid changes in the coordination pattern [21]. The order of the tests was randomized within each experimental session in order to avoid excessive fatigue effects. For the first purpose of the study, the contact time measured by the two devices was considered the dependent variable. Data from the force plate were collected using Cortex software (version 3.6.0; Motion Analysis Corp., Santa Rosa, CA, USA) and imported and analyzed with Excel (Microsoft Corp., Redmond, WA, USA). The contact time was derived considering a 10 N threshold of ground reaction force for initial contact and push-off of the foot with the force plate [19]. The contact time from Optojump system was directly provided by the dedicated software (version, 1.12.15, OptojumpNext, Microgate, Bolzano, Italy). For the second purpose of this study, lateral jumping distance was measured only by the Optojump system to the nearest of 1 cm for the MLRJ (i.e., the distance from the rebounding leg to the starting leg, normalized by each leg length and expressed in arbitrary unit [AU]), and for LCMJ and LSJ (i.e., the distance from the starting leg to the landing leg, normalized by each leg length and expressed in arbitrary unit [AU]). The evaluation of validity of the Optojump system for the measurement of lateral jumping distance was not applicable because of the lack of a criterion measure (i.e., force plate). Participants performed five to seven trials of every test for each leg (alternating one trial for each leg) with a 2 min resting period in between. The five optimal executions were considered suitable for analysis (if participants did not push on the middle of the force plate the trial was not considered appropriate). For the change of direction tests, the trials resulting with the highest and lowest contact time from the force plate were excluded, as well as the corresponding data from the Optojump system. Similarly, for lateral jump tests, the trials resulting with the longest and shortest lateral distance from the Optojump system were excluded, as well as the corresponding data from the force plate.

### 2.3. Statistical Analysis

Data were analyzed using the Statistical Package for the Social Science, version 25.0 (SPSS Inc., Chicago, IL, USA). The level of statistical significance was set at *p* < 0.05 for all computations. Prior to the analysis, the Shapiro–Wilk test was applied to ascertain the normality of data distribution for each trial. Since prior analysis did not detect any gender differences, the sample was combined.

For COD60, COD180, CLRJ, and MLRJ, repeated measures ANOVAs were applied to ascertain the effect of leg (i.e., stance and kicking leg), device (i.e., force plate and Optojump system), and trials (three times for each leg and device) on the dependent variable contact time. For MLRJ, LCMJ, and LSJ, repeated-measures ANOVAs were applied to ascertain the effect of leg (i.e., stance and kicking leg) and trials (three times for each leg) on the dependent variable lateral jumping distance. Effects sizes were calculated as partial eta squared (*ηp*^2^) for ANOVA results.

Pearson product moment correlation was used to assess the strength of the association between the Optojump system and force plate (i.e., the criterion measure) as a measure of concurrent validity for the parameter contact time [22]. Pearson correlation coefficients were calculated considering the average value of the three trials for each leg (i.e., stance and kicking leg). The strength of association was quantified according to the following criteria: ≤0.1 (trivial), 0.1–0.3 (small), 0.3–0.5 (moderate), 0.5–0.7 (large), 0.7–0.9 (very large), and ≥0.9 (almost perfect) [17].

Intraclass correlation coefficients (ICCs) (2,1) and the 95% confidence intervals (CIs) [23] were used for the analysis of internal consistency reliability of the measurement of contact time considering three trials for each leg (i.e., stance and kicking leg) and device (i.e., force plate and Optojump system). ICC and 95% CI values less than 0.5, between 0.5 and 0.75, between 0.75 and 0.9, and greater than 0.90 revealed poor, moderate, good, and excellent reliability, respectively [24]. Absolutely reliability was evaluated with coefficient of variation, standard error of measurement, and Bland and Altman’s 95% limits of agreement methods [22]. Coefficient of variation (CV = (SD/mean)·100) and 95% confidence intervals (CI = (mean ± (2·SE)) were calculated considering the mean, standard deviation, and standard error (SE) of the three trials for each leg and device. Standard error of measurement (SEM) was calculated from the square root of the mean square error term in a repeated measures ANOVA of the three trials. A Bland–Altman plot was used to assess agreement between the force plate and Optojump system plotting the measurement differences (errors) against the respective means [25]. The average value of the three trials for each device was used for the computation of the measurement differences and the respective means. Linear regression analysis was performed considering the measurement differences as dependent variable and the mean as the independent variable. Moreover, the presence of heteroscedasticity or homoscedasticity was detected by calculating the Kendall’s tau (τ) correlation between the measurement differences and the respective means. A τ > 0.1 denotes heteroscedasticity, whilst a τ < 0.1 or negative denotes homoscedasticity [23]. ICC, SEM, and CV were also calculated for the analysis of internal consistency reliability of the Optojump system for the measurement of lateral jumping distance during MLRJ, LCMJ, and LSJ tests.

## 3. Results

### 3.1. Preliminary Inferential Statistic

**Figure 1:**
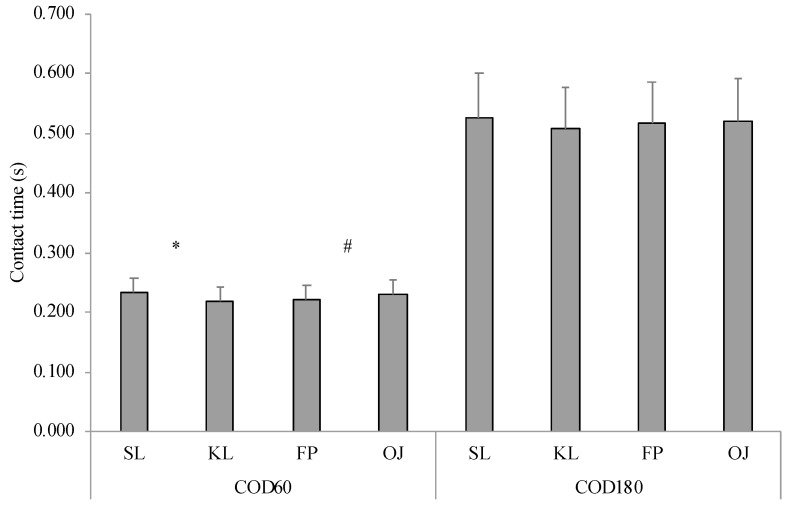
Mean ± SD of contact time for the change of direction tests. COD60: 60° change of direction; COD180: 180° change of direction; SL: stance leg; KL: kicking leg; FP: force plate; OJ: Optojump system. * Main effect of leg (*p* < 0.001). # Main effect of device (*p* < 0.001).

**Figure 2:**
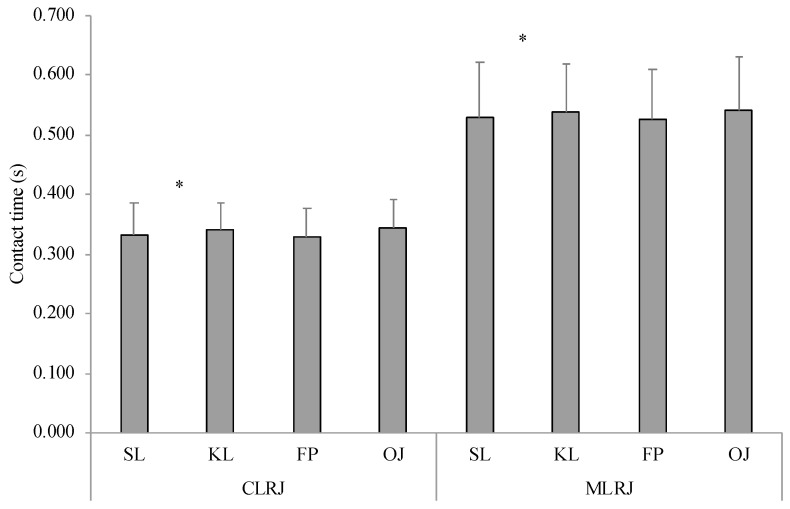
Mean ± SD of contact time for the lateral jump tests. CLRJ: controlled lateral rebound jump; MLRJ: maximal lateral rebound jump; SL: stance leg; KL: kicking leg. * Main effect of leg (*p* < 0.001).

Regarding contact time, repeated measures ANOVAs did not demonstrate interactions among factors for every test. A significant systematic bias (−0.01 s) between the two devices emerged only for COD60 (F_(1,28)_ = 19.379; *p* < 0.001; *ηp*^2^ = 0.401), with the Optojump system (0.230 ± 0.025 s) providing a significant longer contact time compared with the force plate (0.220 ± 0.025 s). A main effect for leg was obtained for COD60 (F_(1,28)_ = 210.721; *p* < 0.001; *ηp*^2^ = 0.879), CLRJ (F_(1,28)_ = 107.302; *p* < 0.001; *ηp*^2^ = 0.787), and MLRJ (F_(1,28)_ = 36.548; *p* < 0.001; *ηp*^2^ = 0.558) showing a different trend. For COD60, a significant longer contact time was found for the stance leg (0.233 ± 0.024 s) in respect with the kicking leg (0.218 ± 0.024 s), whilst for both lateral jumps the kicking leg (CLRJ = 0.341 ± 0.043 s; MLRJ = 0.538 ± 0.081 s) showed significant longer contact times compared with the stance leg (CLRJ = 0.333 ± 0.052 s; MLRJ = 0.529 ± 0.092 s) (Figure 1 and Figure 2). Regarding lateral jumping distance, differences between LCMJ and LSJ emerged for both stance leg (LCMJ = 2.103 ± 0.14 AU; LSJ = 2.029 ± 0.12 AU; F_(1,28)_ = 18.855; *p* < 0.001; *ηp*^2^ = 0.394) and kicking leg (LCMJ = 2.089 ± 0.14 AU; LSJ = 2.011 ± 0.13 AU; F_(1,28)_ = 18.791; *p* < 0.001; *ηp*^2^ = 0.393).

### 3.2. Analysis of Concurrent Validity of Optojump System

Table 1 shows significant (*p* < 0.001) and almost perfect associations (r ≥ 0.95) between the force plate and Optojump system for the measurement of contact time during change of direction and lateral jumping tests. However, a systematic bias was observed for COD60 (*p* < 0.001).

### 3.3. Analysis of Internal Consistency Reliability and Absolute Reliability

The measurement of contact time demonstrated “good” reliability for all conditions except for kicking leg in CLRJ obtained by force plate and in COD180 obtained by Optojump system. Moreover, 95% CI values ranged from “moderate” to “good”, SEM values ranged from 0.008 to 0.032 s, whilst CVs ranged from 10% to 17.5% (Table 2).

**Figure 3:**
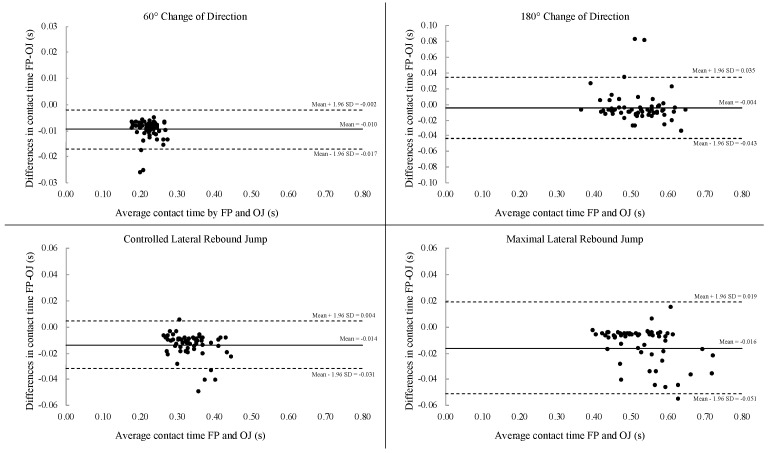
Bland–Altman plots representing comparisons between the force plate and Optojump system for contact time. FP: force plate; OJ: Optojump system. The mean line (solid line) represents the mean difference between the force plate and Optojump system contact time, with the upper and lower lines (dot lines) representing the limits of agreement (Mean ± 1.96·SD).

The Bland–Altman plots show the mean bias between force plate and Optojump system with the limits of agreement for COD60, COD180, CLRJ, and MLRJ (Figure 3). Regression analysis for CLRJ and MLRJ revealed significant B unstandardized coefficient (*p* = 0.021 and *p* = 0.004, respectively), demonstrating the presence of proportional bias. Kendall’s τ correlation coefficients were −0.099 (*p* = 243), −0.097 (*p* = 252), −0.137 (*p* = 107), and −0.171 (*p* = 0.43) for COD60, COD180, CLRJ, and MLRJ, respectively, demonstrating the presence of homoscedasticity.

The measurement of lateral jumping distance showed “good” to “excellent” reliability for MLRJ, LCMJ, and LSJ. Moreover, 95% CI values ranged from “good” to “excellent”, SEM values ranged from 0.032 to 0.055 s, whilst CVs ranged from 6.2% to 7.8% (Table 3).

## 4. Discussion

The main purpose of this study was to investigate the validity and reliability of the Optojump system for testing change of direction and lateral jumping abilities. This study demonstrated for the first time that the Optojump system (OptojumpNext, Microgate) cannot provide a valid measurement of contact time for all the conditions tested, whilst it exhibited a “good” internal consistency reliability for the measurement of the contact time during change of direction and lateral jump tests. Furthermore, for the first time this study proved a “good” to “excellent” internal consistency reliability of the Optojump system for the measurement of the lateral jumping distance. Therefore, these novel findings can further augment the applicability of the Optojump system.

The force plate is always considered the “gold standard” for the evaluation of the jumping ability [11,12,13], even though its application cannot be pursued by researchers and practitioners for every investigation on sport performance. Therefore, the use of alternative systems was deemed necessary in order to enlarge the evaluation with a field-based approach. However, the validity and reliability of any device and experimental procedure should be evaluated and disseminated in order to guarantee valid and reliable measurements. In fact, if standard procedures are maintained, data can be used to detect performance improvements, to compare different groups or conditions, to monitor fatigue and recovery. Since its introduction in 1995, the Optojump system has been tested for its validity versus force plate only considering vertical jump test. Glatthorn and colleagues [13] reported a nearly perfect correlation coefficient of 0.99 between Optojump and force plate when jumping height was evaluated in several vertical jump modalities. Comparably, a nearly perfect correlation coefficient (r = 0.99) for flight time was found in a subsequent investigation [16]. Similarly, Slomka and colleagues [12] found very high correlation coefficients during both countermovement jump (r range = 0.91–0.99) and squat jump (r range = 0.95–0.99) using different algorithms. Recently, Rago and colleagues [17] reported very high correlation coefficients for both flight time (r = 0.98) and jumping height (r = 0.90) in countermovement jump. In accordance with the previous investigations, this study demonstrated the concurrent validity of the Optojump only for the conditions of COD180, CLRJ, and MLRJ, as demonstrated by the almost perfect correlation coefficients. Conversely, the Optojump system did not provide a valid measure for COD60, due to the obtained systematic bias. Similarly, the previous investigations also found a systematic bias between the Optojump and force plate for the parameter flight time [13,16]. Therefore, although being commonly used, the Optojump system might still display a systematic bias compared to the force plate.

Nevertheless, almost perfect correlation coefficients were found between the Optojump system and force plate for lateral jump tests during which mediolateral and anterior–posterior components of force application are also very important [19,26,27]. Therefore, the application of Optojump system for the measurement of contact time during lateral jump tests seems to be less susceptible to bias.

During a change of direction task, the expression of the stretch-shortening cycle is based on the coupling of the two actions, such as deceleration (i.e., eccentric component) and re-acceleration (i.e., concentric component) in the new direction. The temporal action of the change of direction should be minimized in order to exert a powerful movement. Indeed, faster and sharper changes of direction are considered more successful in sport performance [19,28]. However, since a “performance–injury conflict” may be generated, an angle velocity trade-off should be considered [28]. Therefore, the analysis of complex motor abilities, as the change of direction, may require a comprehensive assessment taking into account several factors influencing the performance, such as the technical execution [9,19] and contact time during the directional change. However, caution is required due to possible systematic bias obtained with the measurement of contact time.

The analysis of reliability of the Optojump system and force plate was conducted considering several methods. Differently from previous investigations [12,13,17,29], the present study focused on change of direction and lateral jumping abilities with the measurement of different parameters, such as contact time and lateral displacement. Considering the contact time, the ICC values showed a trend of “good” internal consistency reliability, unless for two conditions. In particular, the force plate and Optojump system demonstrated a “moderate” internal consistency reliability for kicking leg in CLRJ (ICC = 0.730) and COD180 (ICC = 0.697), respectively. Therefore, the findings of the present study did not demonstrate an “excellent” internal consistency reliability of the Optojump system. However, also the force plate, which is considered the “gold standard”, did not show an “excellent” internal consistency reliability. Therefore, caution is required when complex motor abilities are evaluated. It might be speculated that the higher coordinative requirements for the execution of the change of direction and lateral jump tests might cause a higher probability of measurement error.

The choice of using SEM or CV for the interpretation of absolute reliability should be based on the prior analysis for the presence of heteroscedasticity or homoscedasticity [23]. The present data demonstrated to be homoscedastic, therefore SEM method should be preferred. In this study, the SEM values could be considered very low, furtherly demonstrating the good reliability of the Optojump system for measuring contact time and lateral displacement. Combined with the lack of heteroscedasticity, the Bland–Altman analysis demonstrated a small bias of −0.010, −0.004, −0.014, −0.016 s for COD60, COD180, CLRJ, and MLRJ, respectively, being significant only for COD60. Graphical inspection of the Bland–Altman plots (Figure 3) highlights a lower dispersion for COD60 and CLRJ compared with COD180 and MLRJ. However, the regression analysis demonstrated the presence of a fixed bias for COD60 and COD180, whilst a proportional bias for CLRJ and MLRJ, meaning that the measurement of contact time during change of direction tests with the Optojump system might be higher at a constant rate compared with force plate, whilst for the lateral rebound jumps the measurement with the Optojump system might be proportionally higher compared with force plate [30].

Regarding the lateral jumping distance, for the first time this study demonstrated a “good” to “excellent” internal consistency reliability of the Optojump system for the measurement of lateral displacement during lateral jump tests. Indeed, except for stance leg in MLRJ (ICC = 0.872; rated as “good”), all the ICC values were rated as “excellent”, with low standard error of measurements (≤0.055 cm) and coefficients of variation (<10%). Therefore, the findings of the present study on lateral displacement are in line with previous investigations demonstrating an “excellent” reliability of the Optojump system in assessing vertical displacement [13,17,29], fostering the use of the Optojump system for the evaluation of the lateral jump abilities.

In this study, the lateral rebound jumps were performed with a controlled and maximal execution with the aim of assessing the validity and reliability of the Optojump system in measuring performances when athletes have to either control their movement or exert their maximal effort. In fact, not all performances rely on maximal effort and higher postural control and coordinative requirements are necessary when movements are exerted with a controlled effort [31].

It should be noted that different results for validity and reliability may be attributed to the different abilities investigated. The complexity of movement patterns may differ among vertical (i.e., countermovement and squat jump) and lateral (i.e., lateral countermovement, squat, and rebound jump) jumping modalities, and even more when sprinting with different angles of change of direction. The contribution of the three components of ground reaction force could vary during movement patterns executed in different directions [19,32,33]. Furthermore, factors common to all tests (i.e., global) and factors unique to specific tests (i.e., sport-specific) can affect the analysis of reliability of any testing procedure [34].

This study showed a first attempt to investigate the concurrent validity and internal consistency reliability of the Optojump system during change of direction and lateral jump abilities, augmenting the use of the Optojump system for the evaluation of sport-related abilities using a field-based approach, even though a possible measurement error can still be obtained. It is undoubted that the Optojump system proves to be portable, easy to install, and intuitive, hence allowing a broad application in sport performance investigation. Considering all these positive qualities, the Optojump system has been also used as a criterion method when the force plate was not available to assess the validity of other systems [35,36].

The present study has some limitations that need to be addressed and could serve as a guidance for future research. As main limitation, the participants of the present study were representative of a single sport (i.e., basketball) and were an athletic population of collegiate-aged students. Therefore, future studies could explore the reliability and validity of the Optojump system for the proposed change of direction and lateral jump tests also considering other team sports and elite athletes. Moreover, the evaluation of the test–retest reliability was not applicable in the present study since a single testing session for each test has been executed. Even though the findings of this study are in line with those of a previous study design [12], future research could replicate the same experimental procedures but performing two experimental sessions and evaluate the test–retest reliability of the Optojump system. Finally, the validity of the Optojump system for the measurement of lateral jumping distance was not evaluated due to the lack of a criterion measure (i.e., force plate). Therefore, future research for the evaluation of validity could be performed if a long pathway of force plate is available (longer than 2 m).

## 5. Conclusions

The Optojump system can offer a valuable opportunity to measure sport-related performances when a force plate is not available and/or researchers and practitioners pursue a field-based approach in order to detect within-group changes and between-group differences, even though caution is required to avoid misinterpretation of data. Investigating the change of direction ability, the Optojump system can be used to measure the contact time, even though the components of ground reaction force could not be determined, because only a force plate can provide this measurement. When lateral jumping abilities are investigated (with and without a rebound action), the Optojump system can provide a valid and reliable measurement of contact time and lateral displacement. The latter parameter could be measured only in case of large force plate or more than two standard force plates (i.e., 60 × 90 cm). Therefore, the Optojump system is demonstrated to have a valuable application in field-based approach for the investigation of sport-specific abilities in team sports athletes when a force plate is not available.

## Figures and Tables

**Table 1:** Concurrent validity of the Optojump system for the measurement of contact time.

Test	Leg	r	*p*
COD60	SL	0.99	<0.001
	KL	0.99	<0.001
COD180	SL	0.96	<0.001
	KL	0.95	<0.001
CLRJ	SL	0.99	<0.001
	KL	0.97	<0.001
MLRJ	SL	0.98	<0.001
	KL	0.98	<0.001

COD60: 60° change of direction; COD180: 180° change of direction; CLRJ: controlled lateral rebound jump; MLRJ: maximal lateral rebound jump; SL: stance leg; KL: kicking leg; r: Pearson coefficient of correlation.

**Table 2:** Internal consistency reliability of the force plate and Optojump system for the measurement of contact time.

Test	Device	Leg	ICC (2,1)	ICC 95% CI	SEM (s)	CV (%)	95% CI
COD60	FP	SL	0.795	0.663–0.888	0.032	10.5	0.219–0.237
		KL	0.869	0.771–0.931	0.008	11.1	0.204–0.222
	OJ	SL	0.830	0.716–0.908	0.010	10.0	0.229–0.246
		KL	0.887	0.801–0.941	0.008	10.6	0.214–0.231
COD180	FP	SL	0.775	0.633–0.877	0.032	13.4	0.499–0.551
		KL	0.750	0.599–0.861	0.032	13.3	0.483–0.532
	OJ	SL	0.801	0.671–0.892	0.032	14.4	0.502–0.558
		KL	0.697	0.525–0.828	0.032	13.1	0.486–0.535
CLRJ	FP	SL	0.890	0.810–0.942	0.032	15.4	0.308–0.345
		KL	0.730	0.569–0.849	0.032	12.4	0.319–0.349
	OJ	SL	0.875	0.780–0.935	0.032	15.6	0.320–0.359
		KL	0.772	0.629–0.874	0.032	12.7	0.333–0.365
MLRJ	FP	SL	0.871	0.779–0.931	0.032	17.0	0.489–0.554
		KL	0.820	0.701–0.902	0.032	14.7	0.502–0.559
	OJ	SL	0.892	0.813–0.943	0.032	17.5	0.503–0.572
		KL	0.875	0.782–0.934	0.032	15.3	0.516–0.577

COD60: 60° change of direction; COD180: 180° change of direction; CLRJ: controlled lateral rebound jump; MLRJ: maximal lateral rebound jump; SL: stance leg; KL: kicking leg; ICC (2,1): intraclass correlation coefficient (two-way random effects, absolute agreement, single rater/measurement); ICC 95% CI: intraclass correlation coefficient with 95% confidence interval; SEM: standard error of measurement; CV: coefficient of variation; 95% CI: 95% confidence interval.

**Table 3:** Internal consistency reliability of the with Optojump system for the measurement of lateral jumping distance.

Test	Leg	ICC (2,1)	ICC 95% CI	SEM (cm)	CV (%)	95% CI
MLRJ	SL	0.872	0.777–0.932	0.055	7.8	1.879–1.989
	KL	0.911	0.845–0.953	0.045	7.6	1.885–1.993
LCMJ	SL	0.971	0.948–0.985	0.032	6.7	2.051–2.155
	KL	0.972	0.950–0.986	0.032	7.0	2.036–2.142
LSJ	SL	0.912	0.846–0.954	0.032	6.2	1.983–2.075
	KL	0.943	0.899–0.971	0.032	6.9	1.961–2.062

MLRJ: maximal lateral rebound jump; LCMJ: lateral countermovement jump; LSJ: lateral squat jump; SL: stance leg; KL: kicking leg; ICC (2,1): intraclass correlation coefficient (two-way random effects, absolute agreement, single rater/measurement); ICC 95% CI: intraclass correlation coefficient with 95% confidence interval; SEM: standard error of measurement; CV: coefficient of variation; 95% CI: 95% confidence interval.

## Supplementary Materials

The following are available online at https://www.mdpi.com/2411-5142/5/3/55/s1, Graphical representation of the laboratory setting is available as supplementary material with Figures S1–S3.
