# Supplementary material for: Validity and Reliability of a Photoelectric Cells System for the Evaluation of Change of Direction and Lateral Jumping Abilities in Collegiate Basketball Athletes
Source: J Funct Morphol Kinesiol. 2020 Jul 22;5(3):55. doi: 10.3390/jfmk5030055 (PMC7739233; doi:10.3390/jfmk5030055)
Supplement: Supplementary file 1 [file jfmk-05-00055-s001.pdf]

Graphical representation of the laboratory setting

Experimental session for the change of direction tests

During the experimental session two change of direction tests were randomly administered. After the warm up, a minimum of 5 trials were performed for each leg (alternating one trial for each leg) with a 2-minute resting period between trials and a 5-minute resting period between tests in order to modify the test setting.

10 m shuttle sprint with a 180° change of direction at 5 m (COD180)

Participants were required to sprint forward for 5 m, make a COD while on the force plate and sprint for another 5 m. Participants were instructed to push their foot on an area of the force plate 2 near the middle highlighted by an X.

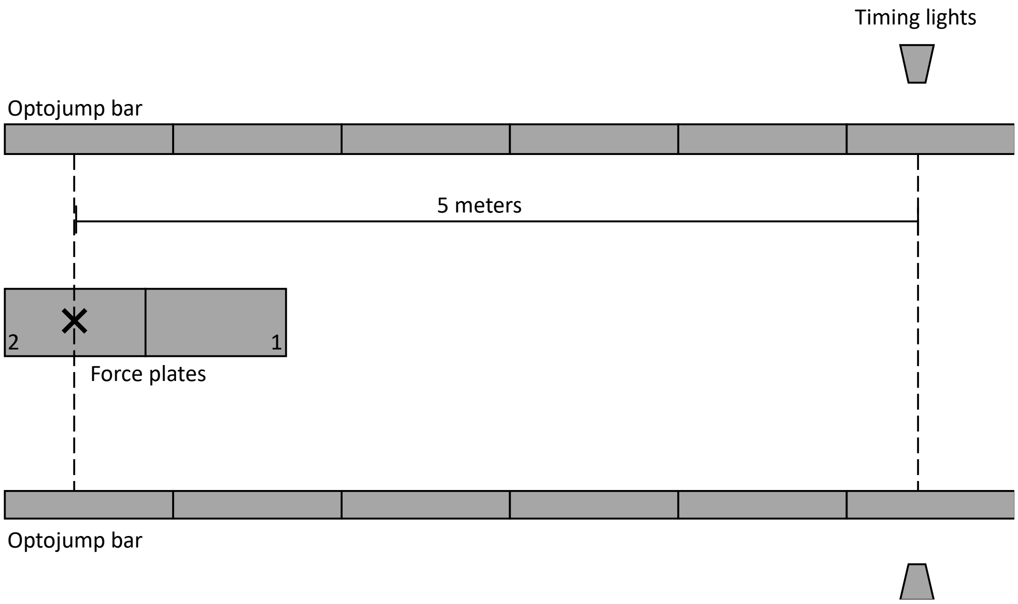

Figure S1. Laboratory setting for the 10 m shuttle sprint with a 180° change of direction

10 m sprint with a 60° change of direction at 5 m (COD60)

Participants were required to sprint forward for 5 m, make a COD while on the force plate and sprint for another 5 m. Participants were instructed to push their foot on an area of the force plate 2 near the middle highlighted by an X.

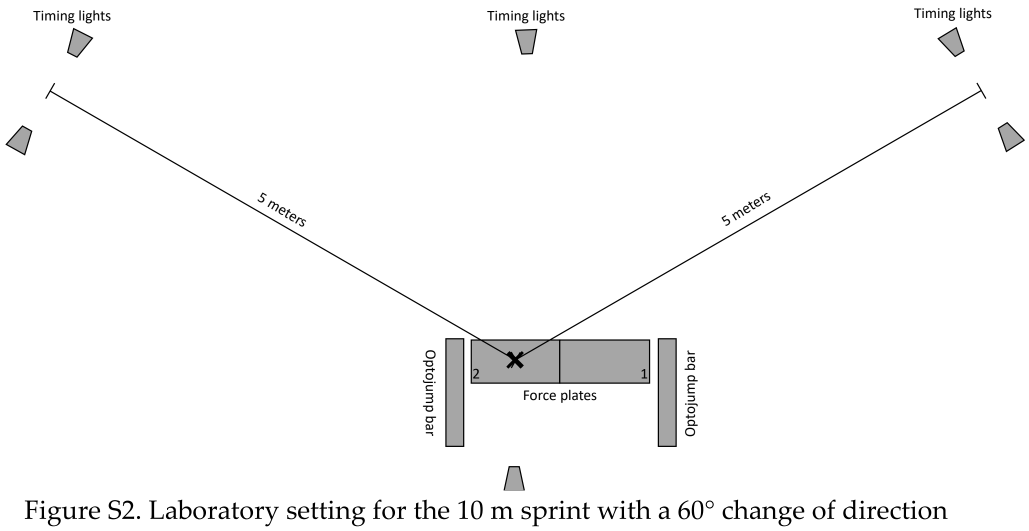

Figure S2. Laboratory setting for the 10 m sprint with a 60° change of direction

Experimental session for the lateral jump tests

During the experimental session four lateral jump tests were randomly administered. After the warm up, a minimum of 5 trials were performed for each leg (alternating one trial for each leg) with a 2-minute resting period between trials and a 5-minute resting period between tests.

Controlled lateral rebound jump (CLRJ) and Maximal lateral rebound jump (MLRJ)

Participants were asked to stand on force plate 1 with a single leg at a distance of 1 m from the middle of force plate 2 keeping the hands on their hips. They were required to jump laterally with one leg (e.g., right leg), rebound (pushing on the force plate 2) with the other leg (e.g., left leg) as fast as possible coming back and landing on the starting point with the starting leg, for CLRJ, or as fast as possible but trying to reach the maximal lateral jumping distance (i.e., as far as possible) landing on the starting leg, for MLRJ. The lateral jumping distance was measured for the MLRJ by the Optojump system to the nearest of 1 cm, considering the distance from the rebounding leg to the starting leg, normalized by each leg length and expressed in arbitrary unit (AU).

Lateral countermovement jump (LCMJ) and Lateral squat jump (LSJ)

Participants were asked to stand on force plate 1 with a single and to keep the hands on their hips. They were required to jump laterally with one leg with and without a countermovement, respectively. For the LCMJ participants were required to bend their knee and self-select the amplitude of the countermovement to avoid changes in the coordination pattern. The lateral jumping distance was measured by the Optojump system to the nearest of 1 cm, considering the distance from the starting leg to the landing leg, normalized by each leg length and expressed in arbitrary unit (AU).

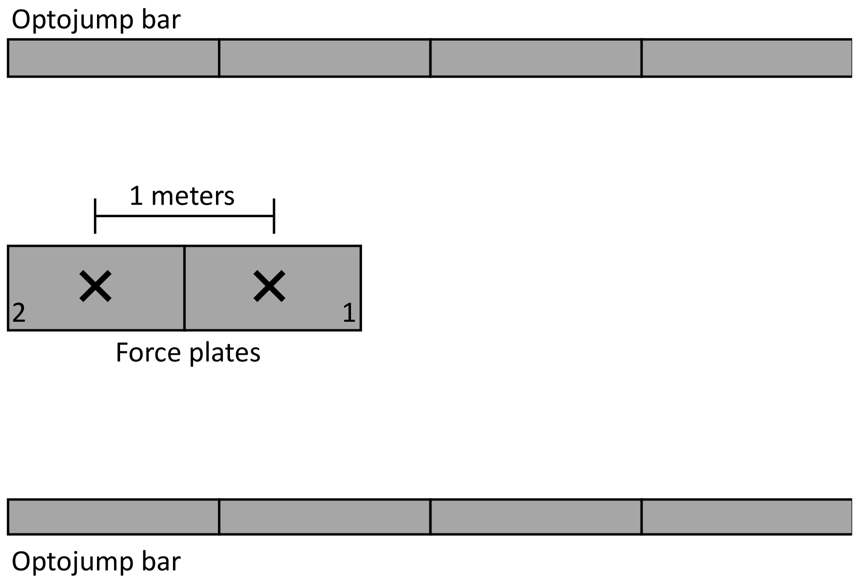

Figure S3. Laboratory setting for the lateral jump tests
